# Patient and Clinician Perspectives on Communication in Primary Care Centres in Qatar—A Focus Group Study

**DOI:** 10.1111/hex.70280

**Published:** 2025-04-25

**Authors:** Nancy Dixon, Liz Cox, Bayan Fraihat, Tareq Khalil Alzeq, Mohammed Abdalla, Nawal Khattabi

**Affiliations:** ^1^ Healthcare Quality Quest Ltd Romsey UK; ^2^ Primary Health Care Corporation Doha Qatar

**Keywords:** focus groups, improvement collaborative, patient‐centred care, patient‐centred communication, patient perceptions and expectations, patient–provider communication, people‐centred care

## Abstract

**Introduction:**

The Primary Health Care Corporation (PHCC) in Qatar manages 31 health centres serving a diverse population of over 1.7 million people. PHCC is fully committed to providing people‐centred care. Patients are routinely asked to complete satisfaction surveys. The surveys have shown continued shortcomings in patients' perceptions of communication with staff. PHCC decided to carry out an improvement collaborative among all centres on improving communication with patients. A realist review was carried out to identify possible communication interventions that improve the outcome of patient satisfaction with communication. Most research studies in the review were carried out in Western countries where patient expectations and experiences may differ. Therefore, focus groups of patients and staff were carried out to learn how patients and staff in Qatar perceive communication in the health centres.

**Methods:**

The purpose of the focus groups was to learn directly how patients and health centre staff experience communication with each other and if the issues experienced could potentially be addressed by the interventions identified in the evidence base. 18 focus groups were carried out, 9 each with patients and multiprofessional staff. Questions were derived from issues raised in research on patient–healthcare professional communication in primary care.

**Results:**

Patients' main concern was how they are welcomed to a health centre, particularly their desire to be greeted with a smile and welcomed courteously. They also discussed confusion about how the health centres work and a lack of understanding of health‐related information. Staff groups also raised the importance of welcoming patients; they also discussed patients' lack of understanding of healthcare subjects. Some issues were consistent with research findings; others were unique to the Qatar setting. Patients focused on their expectations to be seen quickly, whereas staff were aware of the demands on the service and difficulties in meeting patient expectations.

**Conclusions:**

The focus groups identified key issues related to patient–healthcare professional communication in primary care centres in Qatar. These issues were used to set priorities for the improvement collaborative on patient‐centred communication involving all the health centres.

**Patient and Public Contribution:**

PHCC has created and fully implemented a Patient and Family Advisory Group (PFAG), which is a group of patients that closely works with PHCC's leadership to ensure that the patients' voice is heard and that proper collaboration is taking place between patients and PHCC at all levels of its operations (see https://www.phcc.gov.qa/patients-clients/community-engagement). The PFAG was aware of the findings of the patient satisfaction surveys and PHCC's intent to work with patients on improving communication with health centre staff. PHCC also has established a Patient Friends programme in which patients are invited to participate in different activities taking place within PHCC and at each of the health centres. PFAG members and Patient Friends were included among the patients invited to participate in the focus groups. Patients were active participants in half the focus groups. In each of the patient focus groups, the purpose was clearly explained, and patients were invited to add whatever points of discussion they wished to add in addition to the moderator's questions. Patient Friends from all health centres later attended a conference at which findings of the focus groups were openly presented as the basis for the collaborative on improving communication. The Patient Friends then participated equally with staff groups in selecting the patient‐centred communication subjects for improvement via the collaborative and worked as partners with health centre teams on communication improvement projects.

**Clinical Trial Registration:**

Not applicable.

## Summary

- Focus groups with patient and staff groups, which together represent about 70 nationalities, were a useful method for learning how each group perceives communication in primary care settings in Qatar.
- Patients and staff identified many of the same communication issues. Patients were clear about their expectations to be seen and treated quickly, whereas staff groups were aware of the demand on the primary care system and the difficulties in meeting patient expectations.
- The focus groups identified potential subjects for improvement of patient–staff communication, including how patients are welcomed when they arrive at a health centre and for a consultation, and patients' needs to better understand their health concerns and management through asking questions and explaining back to a clinician their understanding of what has been explained to them.

## 1 Introduction

The Primary Health Care Corporation (PHCC) in Qatar manages a network of 31 health centres, catering to a diverse population of over 1.7 million people with more than 4 million health centre visits annually. PHCC is committed to delivering people‐centred care, and its approach has been recognised by Accreditation Canada with the People‐Centred Care Commitment Award [1].

PHCC health centres' patients and staff represent nearly 70 nationalities and speak many languages. This diversity presents unique challenges to communication among patients and staff. Staff training has continuously included communication with patients, and quality improvement projects have been carried out on aspects of communication with patients. Nonetheless, PHCC patient satisfaction surveys consistently revealed serious shortcomings in patient perceptions of the quality of communication between patients and clinical staff.

To address the continuous challenge of negative feedback about communication from patients, PHCC initiated an improvement collaborative involving all primary care centres aimed at enhancing patient–staff communication. At the start of the collaborative, the authors carried out a realist review of research on patient–staff communication in primary care settings. The review identified interventions, such as greeting a patient, encouraging a patient to ask questions about health, using digital health technologies, teach‐back, staff training and quality improvement projects that have been demonstrated to improve the outcome of patient satisfaction, particularly patient perceptions of communication with healthcare professional staff. The review highlighted, however, that most research on communication between patients and clinicians in primary care settings has been carried out in North American and European countries and Australia, and therefore, may not be generalisable to Qatar.

Although PHCC primary care staff were trained in communication skills, it was uncertain if the specific communication interventions identified in the realist review were being used or were suitable for use in primary care settings in Qatar. Therefore, the next stage in the improvement collaborative was to learn more about the communication issues facing patients and staff in Qatar and whether the communication interventions identified by the evidence base would be appropriate for implementation in the PHCC health centre context.

Focus groups have been used widely in the healthcare system in Qatar as a qualitative approach to learning the experiences, perceptions and opinions of people concerned with an issue. Studies that have used focus groups with patients in Qatar have been on: adherence to a Step Into Health programme; meeting the needs of autistic adults; healthcare needs of Arabic speaking primary care patients; self‐management of type 2 diabetes; living with menopause and perceptions of mothers toward oral health services for children [2, 3, 4, 5, 6, 7]. Studies in Qatar using focus groups with healthcare professionals have explored facilitators and barriers to medication error reporting and causes of errors; medical management of older adults; appropriate antibiotic prescription; interprofessional collaboration and perceptions of narcotic use in patients with cancer [8, 9, 10, 11, 12, 13].

Focus groups have also been used to explore communication issues with patients in primary healthcare settings in other countries, including: the use of teach‐back with children; communication with patients with medically unexplained symptoms and communication with patients from different cultures [14, 15, 16]. However, we could not find studies carried out in Qatar or another Middle Eastern country that explored communication issues between patients and staff and possible interventions intended to improve communication.

By focusing on real‐life experiences and perceptions of patients who receive and of staff who deliver healthcare services [17], the focus groups were intended to bridge the gap between work (communication)‐as‐imagined (WAI) and work (communication)‐as‐done (WAD) by staff [18]. The focus groups were aimed at informing the suitability of the communication interventions identified by the realist review and setting priorities for implementation of those interventions that could be valued by patients and staff. By directly involving those most affected by communication practices, PHCC could ensure that any proposed changes in communication practices were contextually appropriate and grounded in the actual needs and preferences of its diverse patient and staff populations.

## 2 Methods

The design and analysis of the focus groups followed the Consolidated Criteria for Reporting Qualitative Research (COREQ) [19].

### 2.1 Purposes and Objectives

The purposes for the focus groups were to learn directly from patients and staff working in the PHCC health centres: (1) how patients and staff experience communication with each other and (2) what patients and staff perceive as issues related to communication that could be addressed by one or more interventions identified from the evidence base.

Objectives for the focus groups with patients and staff were to learn how patients and staff perceive communication with each other with examples of good and ‘could be better’ communication; if patients felt listened to when they were explaining their problems and staff perceptions of listening to patients; patients' knowledge of and attitudes towards digital health technologies and staff support of these technologies with patients; if patients understand what has been explained about a patient's condition and how staff know if patients have understood their explanations; perceptions of the cultures of or languages spoken by staff; and what would improve communication with each other. The objectives were derived from the communication issues addressed by the interventions identified in the realist review.

### 2.2 Number of Focus Groups and Numbers of Participants

To include patients and staff from health centres in all geographic regions in Qatar and to enable health centres in each region to be represented in the focus groups, 9 focus groups with patients and 9 focus groups with staff were conducted. Each focus group was intended to include up to 15 participants.

Patient focus groups included: adult patients from various age groups, Qataris and non‐Qataris, both genders and people who are capable, willing and available to participate in a focus group. Patients who may feel sensitive in front of other people because of their medical condition were excluded from being invited. Staff groups included physicians, dentists, nurses, physiotherapists, pharmacists, laboratory staff, radiology staff, health centre managers, receptionists, customer service staff (called hayyak), administrative staff and regional directors. Patients and staff were solicited to attend the focus groups at the health centre level by members of the PHCC patient engagement team. Arrangements for the focus groups were confirmed by the PHCC patient engagement team with all the participants.

The focus groups were carried out face‐to‐face on consecutive working days in September 2023, with 1–3 focus groups conducted each day. The groups were designed to require 1 h or slightly longer depending on the discussion among the participants, but no more than 1.5 h. The focus groups were scheduled for 1.5 h to allow for arrival and departure of participants.

### 2.3 Protocols

Complete protocols for the conduct of the patient and staff focus groups were developed by the authors and approved by PHCC before the conduct of the groups. The questions and prompts to be used in the focus groups were derived directly from the objectives for each set of focus groups. The questions and prompts were translated into Arabic to be used by the staff moderating the focus groups carried out in Arabic.

### 2.4 Focus Group Moderators and Venue Arrangements

The focus groups carried out in English were moderated by two authors who were trained and highly experienced in carrying out focus groups with patients and healthcare staff. Focus groups carried out in Arabic were moderated by Arabic‐speaking members of the PHCC patient engagement team, trained by experienced moderators. 11 focus groups were in English and 7 in Arabic, 6 with patients and 1 with customer service, reception and administrative staff. None of the moderators had any previous relationship with any of the focus group participants. The focus groups were held in multipurpose rooms in three health centres located in different regions. Participants were seated around tables arranged in a boardroom style.

### 2.5 Recording and Confidentiality Explanation

All focus groups were audio recorded. The recording was explained carefully by the focus group moderators to all participants in each focus group, emphasising that the recording was going to be used only for the purpose of analysis of the content of the discussion in the group and especially that no individual would be identified in any way in the report. Each group was explicitly asked to consent to the recording, and all groups agreed. One group of patients requested that they introduce themselves by name on the recording for the sake of transparency, as explained by the patients; none of the names of patients participating in this group were captured in the data analysis. For all other groups, the participants were invited to introduce themselves before the recording device was switched on, and participants were not referred to by name throughout the recorded discussion.

### 2.6 Processing of the Content of the Focus Groups

All focus groups conducted in English were transcribed using Sonix and Otter.ai software. The accuracy of each transcript was verified by the authors listening to each recording and amending the software‐generated transcript for accuracy as needed. Three software packages for transcribing Arabic to English were tried; none produced transcripts for which the English was completely understandable. Therefore, the Arabic recordings were transcribed by professional Arabic to English translators, with these translators and their employing agency signing a comprehensive confidentiality agreement on the content of the recordings.

The transcripts became the data sources for analysis of the patient and staff focus groups. In addition, notes taken by the moderators during the focus groups were used to identify particularly strong points of discussion or nonverbal communication that could be expressing a view on a subject.

### 2.7 Analysis of the Transcripts of the Focus Groups

The framework method for the analysis of qualitative data was used to analyse the focus group transcripts [20]. Transcripts of the interviews were broken down by protocol question. Two of the authors independently read the content relating to each question in the focus groups and labelled the responses, which became codes. Codes used were to identify generally positive or negative comments about communication with staff or with patients and keywords or phrases expressed by participants in the focus group that elaborated on the positive or negative comments or reflected dominant experiences or opinions about an aspect of communication. Codes were extended for subjects for which there was extensive discussion. The analysts discussed instances of disagreements on the coding of transcript content and resolved any differences. A spreadsheet was used to summarise by codes the themes of responses to each of the focus group questions, as well as any noteworthy comments.

Although the moderators of the focus groups asked the questions directly related to the focus group objectives, the patient groups tended to expand the scope of the focus groups to include their concerns and experiences about using the health centres. The additional subjects raised by the patients were also included in the analysis.

## 3 Patient Focus Group Results

Eight of the patient focus groups represented patients from different regions in Qatar. The ninth group was made up entirely of members of the PHCC Patient and Family Advisory Group (PFAG). In total, of 136 patients invited, 65 patients participated in the 9 focus groups, with between 4 and 11 patients in each group. At the start of most patient focus groups, patients spontaneously expressed their gratitude for the availability of and free access to the health centres and for being invited to contribute their ideas about communication between patients and staff working in the health centres. Many patients praised the services they had received from a health centre and the way some staff working in a health centre treated them.

Patients responded to the moderator questions about communication with staff in the health centres. However, they also brought up problems they experienced with getting information on using the health centres, for example, how to renew a health card, get an appointment with a hospital specialist or obtain medications for complex conditions, because not every health centre stocks all medications.

### 3.1 Patients' Perception of Communication Issues With Health Centre Staff

Eight themes were derived from the analysis of the patient focus groups.

***Patients' theme 1—Wanting to be welcomed or greeted on arrival—*** How patients are received at the health centres was the subject that the patient groups talked about most. They emphasised the importance to patients of being welcomed and greeted on arrival at a health centre, with staff making eye contact with a patient, smiling, and speaking with a patient in a kind way. The patients made a point about Qatar being an Islamic country and that it is in the culture to be welcoming and hospitable, and they wanted this culture to be demonstrated in the health centres.

***Patients' theme 2—Not sharing clinical information with nurses—*** Patients don't want to divulge clinical information in physical spaces in a health centre where others can overhear their information. The spaces where patients are triaged by nurses are not sufficiently private for the patients. However, nurses perceived that patients don't want to share clinical information because they don't respect the nurses.

***Patients' theme 3—Not knowing where to go—*** Patients said they don't always know where to go for a service in a health centre, and they may walk around the health centre looking for the room they are supposed to go to. Sometimes they just open a physician's closed door to confirm that they are in the right place.

***Patients' theme 4—Some doctors and some nurses are not good at communication with patients—*** Patients commented that some staff don't make eye contact with the patient, don't explain, don't listen or don't want the patient to ask questions.

***Patients' theme 5—Not always understanding the information they are given by a healthcare professional—*** Patients explained that they do not always understand the information being explained by a physician or another member of staff, particularly about their medications.

***Patients' theme 6—The language barrier—*** Patients discussed what happens when the patient and the physician don't speak the same language, specifically that Google Translate is not helpful, and they don't want staff interpreters because of their concerns about privacy and confidentiality.

***Patients' theme 7—Preferences for digital health technologies—*** Patients would like the PHCC to ask patients which digital health technologies they prefer to use, and then for PHCC to use those technologies with a patient.

***Patients' theme 8—Communication is a management issue—*** The PFAG specifically made the point that communication is a management issue, and problems about communication should be addressed by the health centre manager.

The patients' key themes are further explained in Table 1.

**Table 1:** Patient perceived communication issues with health centre staff.

Communication issue	Explanation
Patients don't always feel they are welcomed, helped and respected by the reception and greeter function at the health centres.	Some of the staff who receive patients when they arrive at some health centres do not smile at patients, may not look at the patient when dealing with him/her, may not greet or welcome the patient, may give some patients priority in the queue system, or talk on their mobile phones and tell the patient they are busy. Patients may contribute to less than ideal communication because they are in pain and may not interact with staff as they normally would.
Patients don't want to tell the nurses why they have come to the health centre.	The space where the nurse sees the patient in some health centres is not sufficiently private, and someone outside the area can hear the conversation. Sometimes, staff leave the door open, and people sitting outside can hear everything.
Patients don't always know where to go and where to wait for each room number; they requested a map of room numbers for the health centres. Some patients don't wait to be invited into a physician's consulting room and walk into the room as soon as the last patient exits.	Patients sometimes knock on physician's consultation room doors when the physician is with a patient just to learn if they are waiting in the right location. Also, patients don't know that the physician needs to clean the room (paper on the bed from the last patient and supplies used with the last patient discarded safely), enter information about the last patient's condition and management plan into the IT system, and retrieve and scan the record for the next patient in preparation for seeing the patient.
Patients perceive that some physicians are not good communicators. Patients perceive that some triage nurses seeing walk‐in patients don't communicate well with patients.	The physicians whom patients see as not good communicators don't make eye contact with the patient, don't explain the patient's condition or why tests are being requested, don't really listen to the patient (possibly because they are pressed for time), or don't seem to like the patient asking the physician questions. When the physician lacks communication skills, the patient often returns to a health centre to see another physician and get another opinion. The nurses who don't communicate, according to the patients, don't smile or interact with the patient before taking the patient's vital signs.
Some patients aren't clear about instructions for taking their medications or why a medication is being changed or stopped.	The physician or the pharmacist does not always explain to the patient why a medication has been prescribed, how to use a prescribed medication, or why a medication has been changed or stopped. In some health centres, there is no space to have a confidential conversation with a pharmacist or a pharmacy counselling room.
Patients refer to using Google Translate when a physician and the patient don't speak the same language, but patients acknowledged that there could be problems with using Google Translate. Patients may not always welcome having interpreters when the physician or nurse doesn't speak their language.	When a physician or nurse doesn't speak the patient's language, there is a risk of miscommunication about the patient's symptoms, and if an interpreter is used, the patient feels s/he is losing privacy and confidentiality about his/her condition.
Patients would like to be asked their preference for the media to be used to communicate with them, for example, a patient portal, text, WhatsApp or app.	Patients vary in the way they use the media and would prefer to use the medium they are used to.
Patients, especially the Patient and Family Advisory Group, see communication as a management issue.	If communication is a priority for top management, the organisation will consider how to address issues that affect communication such as the number of patients seen by a physician in a day and facility constraints on communication between patients and staff.

### 3.2 Other Issues Raised by Patients

In addition to issues related to communication with health centre staff, the patients brought up systems issues relating to getting fast appointments with a physician, registering and obtaining a health card, understanding how the ‘walk‐in’ (urgent care) system works (that a patient triaged as not urgent may be given an appointment to be seen later), and lengthy waiting to see a physician or get a medication in a health centre. They also discussed the mixture of roles of health centres, trying to be both family medicine centres and urgent care centres, and the fact that the patients miss the experience of the family medicine model. The patients also expressed some confusion about how the healthcare system in Qatar works, for example, that patients can't book an appointment with a hospital specialist through the call centre that handles appointments at health centres and that such an appointment needs to be a referral from a health centre physician.

The patients commented on the Arab and Middle Eastern culture in which people speak directly to the point, expressing what they want. They were candid in discussing their expectations for the provision of healthcare services. They said: ‘We want everything, and we want it now.’ Many staff members interpreted the patient's approach as: ‘Shouting is the patient's language.’

## 4 Staff Focus Group Results

Of 130 staff members invited, 76 staff participated in the focus groups. The groups were organised by staff category: physicians and dentists (27 attending three groups); nurses and allied health professionals (18 attending two groups); pharmacists (9 attending one group); radiology and laboratory staff (7 attending one group); admin, customer service and reception staff (7 attending one group); and regional directors, health centre managers and leads (8 attending one group).

### 4.1 Staff Perception of Communication Issues With Patients

Staff participating in the focus groups often described how they attempt to communicate with patients using communication skills. However, they acknowledged the communication problems experienced with patients. Eight themes were identified from the staff focus groups.

***Staff theme 1—Communication skills staff should apply—*** Physicians in the focus groups described communication with patients as a skill with principles to follow and develop through practice. They described communication as the most important part of the dialogue between a clinician and the patient and as a part of treatment. They referred to the patient expecting the physician or dentist to be a good listener without interrupting the patient. They also described setting an agenda with the patient at the beginning of the consultation, learning all the issues the patient is raising and negotiating with the patient about how the patient's problems will be handled in the consultation. The physicians acknowledged that communication with a patient takes time, which may be hard for physicians when they are expected to see many patients in a shift. They also stated that improving the patient experience with better communication required all staff working in the health centre, not just the physicians or dentists.

Nurses and physiotherapists also discussed the importance of smiling, greeting the patient, listening to the patient and trying to make the patient comfortable, getting up from their chair to open the door and welcome the patient, as well as providing information for the patient and using open‐ended questions to get the patient to talk about his or her concerns. Regional and health centre managers discussed the importance of greeting and welcoming patients to a health centre, with staff members introducing themselves to patients and making eye contact with patients.

***Staff theme 2—Patients not always understanding the information they are given by a healthcare professional—*** Pharmacists discussed the importance of teaching the patient about the medication prescribed, including why it has been prescribed, for how long and what could happen if the patient stopped taking the medication, and being sure that the patient has understood the pharmacist's explanation. Pharmacists also pointed out that communication with a patient takes time, and sometimes the pharmacy is so busy, there isn't time to have this type of communication with each patient. They also commented that many patients do not want to engage with the pharmacist about their medications.

Laboratory and radiology staff referred to sources of misunderstanding by patients about their services, such as the need for patients to complete a required preparation for some investigations or that not all investigative services can be carried out in every health centre and that patients may have to travel to another health centre for some investigations. The admin, customer service and reception staff also discussed the importance of patients' understanding how the health centres work and having access to information about the health centres' administrative systems.

Staff acknowledged that they may not explain information in a way the patient understands, and the patient can make a return visit to a health centre or go to another member of staff to ask questions.

***Staff theme 3—Patient lack of understanding of the health centres and staff roles—*** The physicians and managers both discussed the patient's lack of understanding of the role of PHCC and the health centres, citing that they overhear patients talking on their phones and telling people they are at the hospital. They discussed the confusion that has emerged from adding urgent care to the health centres, when the previous model had been to deliver family medicine with families having a designated physician responsible for their care, and that the urgent care concept now dominates the way the health centres tend to be working and physicians can't maintain continuity of care with their patients.

The healthcare professional staff groups all commented that patients don't seem to understand the professionals' roles and that patients tend to have a highly simplified view of what these staff actually do in their jobs. For example, laboratory staff expressed that patients think that all they do is collect blood and put it into a machine; the patients ask the staff directly for the results from the machine. Pharmacists said that patients think that all pharmacists do is take medications from the shelves and give them to the patient. Physiotherapists commented that patients do not understand that much of what a physiotherapist does is teach patients to manage their own conditions.

***Staff theme 4—The language barrier—*** All the staff groups acknowledged the communication difficulties when staff members and patients don't speak the same language. Staff try to find a member of staff who speaks the patient's language to help; however, the physicians mentioned that staff serving as interpreters need to be trained for this role. They also mentioned that patients seem to value staff members differently, depending on the nationalities of the patients and staff, and that it is valuable for staff to know the social norms for the nationalities of patients they treat, for example, culturally acceptable and unacceptable gestures.

***Staff theme 5—Patients not knowing where to go—*** Staff acknowledged that patients are sometimes sent to the wrong location upon arrival at a health centre. When patients want an immediate appointment, the call centre may direct patients to a health centre they have not previously attended and therefore, the patients don't know where to go. Also, the call centre can send patients to a health centre that does not offer the service the patient wants, for example, a dental appointment or an investigation not carried out in all health centres.

***Staff theme 6—Patients not sharing information with nurses—*** Patients don't want to disclose their personal details, saying it is all in the patient's record, and they don't know why nurses are asking them to verify their identity. Also, they don't want to give clinical information to nurses carrying out triage for urgent care, or they give different versions of their symptoms to ‘pass’ triage and get to see a physician quickly.

***Staff theme 7—Teamwork among staff—*** The staff groups also made the point that communication among staff members affects the quality of communication with patients. They referred to the need for all staff groups working in a health centre to improve their communication with each other, especially across nationalities and services. The intent expressed was to work as a team delivering care to patients, specifically improving communication among staff groups about the patient ‘pathway’ in a health centre; patient demands for clinically inappropriate services; services or medications not available at all health centres; or referrals for inappropriate therapies for some patients.

***Staff theme 8—Management support—*** Staff groups referred to the need for management support to facilitate communication between patients and staff, particularly for management to take a balanced approach when a patient complains and to support staff when they follow PHCC policies.

Further explanations of the issues staff groups raised about communication between patients and health centre staff are in Table 2.

**Table 2:** Staff perceived communication issues with patients.

Communication issue	Explanation
Staff should apply communication skills in consultation with patients.	Communication skills should include welcoming the patient, listening to the patient's concerns, negotiating with the patient on how the patient's concerns will be handled and explaining the management plan to the patient.
Some physicians don't fully explain the patient's health condition or management plan.	Physicians may lack communication skills, may not speak the same language as the patient or may be pressured for time because of the number of patients to be seen.
Patients don't talk with the pharmacist about medication.	Some patients don't like to talk to the pharmacist, or there is a lack of time or a language barrier, or the pharmacist lacks communication skills, or there is no space to have a confidential conversation with a pharmacist.
Patients make unnecessary quick return visits to a health centre or go to a nurse or pharmacist in the same visit to ask questions.	Patients didn't understand the information provided by the member of staff, perhaps because of speaking different languages, the clinician involved didn't have time to confirm patient understanding of an explanation, the patient didn't trust what the clinician seeing the patient said or the patient information available electronically is only in English.
Many patients don't understand that if they attend an urgent care service, they will be triaged and if their condition is not urgent, they will be given an appointment later.	Patients expect to see a physician immediately; they don't understand why triage is important and they don't accept being given an appointment later when they want to be seen now (often for sick leave authorisation).
Some patients attending the urgent care service don't want to tell the nurses why they have come to the health centre, or they give different versions of their symptoms to different staff members.	Sometimes there is only a curtain separating patients. Some patients do not want to inform the nurse about the reason for their visit because they want to be seen immediately by a physician and not go through the triage process. Patients give different versions of their symptoms to get through the triage process and be seen quickly by a physician.
Some patients don't complete the required preparation for some investigations, such as bowel preparation or fasting.	Some patients say the doctors did not explain the preparation, and they may not agree to complete the required preparation (which may result in clinically invalid results and require a repeat investigation)
Some staff are unsure what the patient needs or don't understand what the patient is trying to explain.	Patients and some staff, especially nurses, speak different languages.
There can be miscommunication by staff with patients when they arrive at a health centre or lack of direction for the patient.	Patients are sometimes misdirected on arrival to the wrong services or don't know where to go in the health centre or where to wait for the service they have come for.
Patients attend health centres where the staff have no previous experience with the patients and where the patients are not registered.	The call centre, 107, may direct patients to a far‐away health centre if patients want a quicker appointment, or the call centre can misdirect patients for dental care or for an investigative service to a centre that does not provide the service.
Patients don't want to disclose their name and date of birth, and some patients are offended when staff ask.	Patients say it is in their records, and they don't understand why staff need to check patient identification every time.
Patients use the complaints system to get what they want.	Patients' expectations are to get what they want quickly, and they have learned that making a complaint will result in getting what they want.
Staff need to work together to improve communication with patients.	All staff are involved in communication with patients, and teamwork is needed to make improvements.
Staff perceive that management support for staff would influence their communication with patients.	Staff often feel too busy with too many patients to communicate properly with each patient, and staff morale is affected by the workload. Also, staff perceive that management always supports patients when they complain.

## 5 Discussion

In the focus groups with patients and staff, it is notable that many of the communication‐related themes raised by patients and staff were the same or highly similar, although they were expressed somewhat differently by patients and staff, as summarised in Table 3.

**Table 3:** Communication‐related similar themes discussed by both patients and staff.

Theme	Patients' way of expressing	Staff members' way of expressing
Welcome and greeting	Patients don't always feel they are welcomed, helped and respected by the reception and greeter function at the health centres. Patients don't always know where to go and where to wait for each room number; they requested a map of room numbers for the health centres.	There can be miscommunication with patients when they arrive at a health centre or a lack of direction for the patient. Patients attend health centres where the staff have no previous experience with the patients and where the patients are not registered.
Patients' sensitivity about discussing health problems or medications with non‐physician staff	Patients don't want to tell the nurses why they have come to the health centre. Patients perceive that some triage nurses seeing walk‐in patients don't communicate well with patients.	Some patients attending the urgent care service don't want to tell the triage nurse why they have come to the health centre, or they give different versions of their symptoms to different staff members. Patients don't want to disclose their name and date of birth, and some patients are offended when staff ask.
Patients don't always understand what has been explained to them	Patients perceive that some doctors are not good communicators. Some patients aren't clear about instructions for taking their medications or why a medication is being changed or stopped.	Some doctors don't fully explain the patient's health condition or management plan. Patients make unnecessary quick return visits to a health centre or go to a nurse or a pharmacist in the same visit to ask questions. Patients don't talk with the pharmacist about medication. Some patients don't complete the required preparation for some investigations, such as bowel preparation or fasting.
The language barrier	Patients refer to using Google Translate when a physician and the patient don't speak the same language. Patients may not welcome having an interpreter when the doctor or nurse doesn't speak their language.	Some staff are unsure what the patient needs or don't understand what the patient is trying to explain [because of speaking different languages].
Communication as a management issue	Patients, especially the Patient and Family Advisory Group, see communication as a management issue	Staff often feel too busy with too many patients to communicate properly with each patient, and staff morale is affected by the workload. Also, staff perceive that management always supports patients when they complain.

A clear issue that emerged from both the patient and staff groups was about patient expectations and the implications of patient expectations for the staff's delivery of services. Patients were clear that they wanted to be seen quickly by a physician in a health centre, and any delays in getting appointments were enormously frustrating to patients. Patients had various strategies for getting around the delays, such as coming to a health centre as a ‘walk‐in’ patient and then ‘making a fuss’ if the triage nurse determined that they did not need to be seen urgently and could be given a routine appointment, or making several calls to the call centre to get an appointment, or taking an appointment at a different health centre from the one where they are registered just to have a quick appointment.

On the other hand, staff were frustrated that the urgent care service was tending to dominate the daily workload for clinical staff and that it was challenging to maintain family medicine services when physicians needed to see patients presenting for urgent care. Staff were deeply concerned that when some patients are not seen immediately, even when they have minor self‐limiting illnesses or they just wanted a ‘check‐up’, the patients turn to the health centre's complaint system to achieve their objectives. Staff can feel defensive about making clinically appropriate decisions for patients when the complaint system is used in this way by patients.

Some of the themes in the focus groups are consistent with the barriers to interprofessional collaboration identified among healthcare professionals in primary care centres in Qatar, specifically that patients are reluctant to provide information to healthcare professionals other than the physician; they see the physician as ‘the pinnacle health professional’; and they want to be mainly seen and examined by physicians. In the same study, staff participants perceived a lack of understanding of other healthcare professionals' scope of practice. In addition, they reported concerns about the prevalence of a ‘blame culture’ when something is seen as going wrong [12].

Nonetheless, these focus groups were helpful in pointing to directions for improvement of communication between patients and health centre staff. In addition to training all staff to acquire and apply effective communication skills such as maintaining eye contact, smiling and listening, specific projects for improvement could be identified as needed from the themes in the focus groups. These projects included welcoming and greeting patients; encouraging patients to ask questions about their health and answering the questions; and doing more to help patients understand the health‐related information about their care, which staff were providing to patients through the intervention of teach‐back. This analysis of the key communication‐related issues led PHCC to involve all the health centres in these three improvement projects: welcoming and greeting patients to the health centre and to every service in the health centre; encouraging patients to ask questions about their health and management plans; and using teach‐back to confirm that when patients are given information about their condition and their management plans, patients fully understand the explanations.

Some of the patients' behaviours, identified by both the patients and staff, concern a lack of understanding of the primary care health system, management of patients' health conditions and treatment, including using medications as prescribed. The concept that underpins these issues is health literacy. The level of health literacy in the Qatari population does not seem to be known; however, basic or limited health literacy levels of 21% in the United States [21], 43% in the United Kingdom [22], and 47.6% in European Union countries [23] are unlikely to be exceeded in Qatar, given the diversity of the population. The improvements in encouraging patients to ask health‐related questions and teach‐back are intended to achieve the outcome of improved patient perception of communication in the primary care centres because these interventions are addressing the underlying context of low health literacy in primary care centres in Qatar. The intended improvement of welcoming patients reflects the patients' perception of the Qatari culture and reminds staff to treat patients with kindness.

## 6 Conclusions

Focus groups with patients and primary care centre staff in Qatar identified key issues related to patient–healthcare professional communication in health centres in Qatar. Several issues were shared between the patients and staff, including the importance of welcoming and greeting patients in every service in a health centre; patients' lack of understanding of how the health centres work, especially in the delivery of urgent care; patients don't always understand what healthcare professional staff explain to them; and the importance of patients' sensitivity about discussing their health problems or medications with various healthcare professional staff. The issues related to welcoming a patient, encouraging patients to ask questions and using teach‐back to ensure that patients understand what has been explained to them were identified as subjects for the improvement collaborative on patient‐centred communication involving all the health centres.

## Author Contributions

**Nancy Dixon:** conceptualisation, investigation, formal analysis, methodology, writing – original draft. **Liz Cox:** data curation, investigation, supervision, validation, writing – review and editing. **Bayan Fraihat:** investigation, project administration. **Tareq Khalil Alzeq:** investigation. **Mohammed Abdalla:** investigation. **Nawal Khattabi:** conceptualisation, writing – original draft.

## Ethics Statement

The focus groups were designed to be consistent with the *Guide to Managing Ethical Issues in Quality Improvement or Clinical Audit Projects*, https://www.hqip.org.uk/wp-content/uploads/2017/02/guide-to-managing-ethical-issues-in-quality-improvement-or-clinical-audit-projects.pdf, which is UK national guidance on this subject. The entire protocol for the focus groups was reviewed and approved by the Patient Engagement Department of the Primary Health Care Corporation.

## Consent

All patients consented to participate in the patient focus groups before each focus group began.

## Conflicts of Interest

The authors declare no conflicts of interest.

## Data Availability

Transcripts of the focus groups and data analysis tables are available from Healthcare Quality Quest (contact Liz.Cox@hqq.co.uk) for researchers who meet the criteria for access to confidential data.
